# Detection of Leak-Induced Pipeline Vibrations Using Fiber—Optic Distributed Acoustic Sensing

**DOI:** 10.3390/s18092841

**Published:** 2018-08-28

**Authors:** Pavol Stajanca, Sebastian Chruscicki, Tobias Homann, Stefan Seifert, Dirk Schmidt, Abdelkarim Habib

**Affiliations:** Bundesanstalt für Materialforschung und—Prüfung (BAM), Unter den Eichen 87, 12205 Berlin, Germany; sebastian.chruscicki@bam.de (S.C.); tobias.homann@bam.de (T.H.); stefan.seifert@bam.de (S.S.); dirk.schmidt@bam.de (D.S.); karim.habib@bam.de (A.H.)

**Keywords:** distributed acoustic sensing, DAS, distributed vibration sensing, DVS, fiber-optic sensors, pipeline monitoring, leak detection, pipeline vibrations

## Abstract

In the presented work, the potential of fiber-optic distributed acoustic sensing (DAS) for detection of small gas pipeline leaks (<1%) is investigated. Helical wrapping of the sensing fiber directly around the pipeline is used to increase the system sensitivity for detection of weak leak-induced vibrations. DAS measurements are supplemented with reference accelerometer data to facilitate analysis and interpretation of recorded vibration signals. The results reveal that a DAS system using direct fiber application approach is capable of detecting pipeline natural vibrations excited by the broadband noise generated by the leaking medium. In the performed experiment, pipeline vibration modes with acceleration magnitudes down to single μg were detected. Simple leak detection approach based on spectral integration of time-averaged DAS signals in frequency domain was proposed. Potential benefits and limitations of the presented monitoring approach were discussed with respect to its practical applicability. We demonstrated that the approached is potentially capable of detection and localization of gas pipeline leaks with leak rates down to 0.1% of the pipeline flow volume and might be of interest for monitoring of short- and medium-length gas pipelines.

## 1. Introduction

Pipelines are an important part of the world’s material and energy supply infrastructure. Various pipelines ranging from local industrial piping systems to global pipelines traversing continents are in use around the world. As the world’s pipeline infrastructure grows and ages, regulatory requirements on pipeline safety and reliable operation are increasing as well. Implementation of a robust and reliable leak detection system still remains one of the core issues of pipeline condition monitoring [1]. Origin and signature of pipeline leaks can differ considerably depending on a number of factors including transported medium, pipeline type and operation conditions. Therefore, pipeline leak detection systems typically need to be tailored and verified for the individual application cases. There are a number of different technologies that can be used for pipeline leak detection [2,3,4,5]. They can be broadly divided into two categories; internal and external monitoring methods, sometimes also referred to as software- or hardware-based systems, respectively. Internal systems utilize different computational approaches to infer the occurrence of a leak from pipeline internal parameters such as product pressure, temperature, density, or flow rate. These types of systems are typically cheap and easy to implement as they rely on processing data from sensors that are either already part of the pipeline or can be easily retrofitted. However, their performance is typically limited in terms of minimum detectable leak size, speed of leak detection and precision of leak localization. External systems rely on using additional sensors along the pipeline to detect the external manifestations of a possible leak, e.g., acoustic signals, temperature changes or presence of chemical spill. External systems have typically higher hardware and installation costs but may potentially offer more reliable and faster detection with high localization accuracy even for smaller leaks.

In the recent years, distributed fiber-optic sensors (DFOS) have been attracting increasing attention in the pipeline industry for various monitoring tasks [6,7,8,9]. DFOS rely on different types of light backscattering in optical fibers, based on which they can be broadly split into three categories; Brillouin- [10], Raman- [11], and Rayleigh-based [12] DFOS systems. Depending on the sensing principle, DFOS may provide distributed information on local attenuation, temperature and strain along tens of kilometers of the fiber length. Using DFOS as external pipeline monitoring systems is very attractive since a single interrogation unit can provide spatially- and temporally-continuous monitoring of extended pipeline lengths. Various DFOS schemes have been proposed for pipeline leak detection over the years. These include attenuation-based sensors using special hydrocarbon-reactive fiber cables [13,14], interferometric-based systems detecting leak acoustic emission [15,16,17], or Raman- and Brillouin-based systems relying on detection of leak-induced temperature gradients in the vicinity of a pipeline [8,9,18].

Distributed acoustic sensing (DAS), sometimes referred to also as distributed vibration sensing (DVS), belongs to the latest developments in the broad range of available DFOS techniques. DAS systems belong to Rayleigh-based DFOS family, relying on a principle called coherent optical time-domain reflectometry (C-OTDR) [19]. In the simplest C-OTDR form, short coherent laser pulses are sent into the fiber where they are partially back-reflected by local scattering centers originating from sub-wavelength refractive index inhomogeneities formed during the fiber drawing. Weak backscattered light is detected at the fiber input end at high acquisition rate. Recorded time evolution of backscattered light intensity of a single pulse contains information on immediate configuration of local backscattering centers along the fiber. Position along the fiber can be inferred from the pulse relative time-of-flight in the fiber and the detected local intensity is a product of light interference from scattering centers encompassed in the pulse envelope. Scatterrer distribution is an individual characteristic of a particular fiber. However, even small perturbations of a local scatterer distribution due to external perturbations, such as strain or temperature changes, may lead to significant variation of recorded backscattering intensity profile. These changes of fiber backscattering profile between consecutive pulses can be monitored at high repetition rates, thus providing highly-dynamic information on temperature or strain changes along the optical fiber. Such a highly-dynamic strain monitoring lies in the core of the DAS technology [20]. Above-described single-pulse direct-detection C-OTDR represents the simplest implementation of the DAS technology, sometimes also referred to as amplitude-based DAS. In fact, designation DAS is a misnomer in this case, as the system can measure neither phase nor true amplitude of an acoustic/vibration signal. Some researchers and manufacturers use more appropriate DVS designation for these types of systems allowing only qualitative distributed vibration measurement. Nevertheless, numerous more sophisticated configurations of the system targeting true quantitative dynamic strain measurement have been proposed over the recent years [21,22,23,24,25,26], some of which are also available commercially.

Being able to detect highly-dynamic vibration signals up to the kHz range, DAS systems have piqued the interest of pipeline industry with their high potential for pipeline monitoring tasks. Most typically, the systems are being considered and employed for detection of third-party intrusion on the pipelines [8,27,28,29,30]. Nevertheless, utilization of DAS for pig tracking [29,31,32], flow monitoring [33,34], and leak detection [35,36,37,38] has been explored as well. In a majority of applications, sensing optical cable is laid along the pipeline in its close vicinity. Leak monitoring is based on the detection of either negative pressure wave associated with leak opening or broadband leak-induced acoustic noise. However, signal transfer from the pipe to remote fiber is not ideal and the leak detection is typically limited to relatively large leaks. Direct fiber application to the pipe may provide better signal transfer and higher DAS system sensitivity for detection of even small leaks (<1%) [39]. Here, we present the results of measurement campaign investigating feasibility of detection of pinhole leaks in gas pipelines using DAS with fiber applied directly to the pipe. We show that using this approach, even weak leak-induced natural vibration modes of the pipeline can be detected and used as pointers for pipeline leak localization.

## 2. Materials and Methods

### 2.1. Model Pipeline System

**Figure 1:**
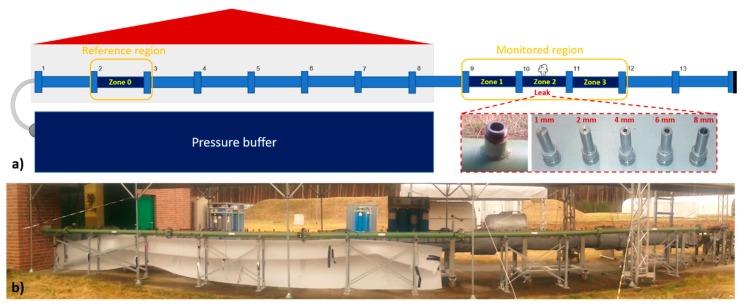
(**a**) Schematic illustration of the used pipeline system highlighting pipe zones instrumented with optical fiber. Inlay: pipeline side adapter and holey adapter caps used to simulate the leak in the pipeline; (**b**) Photo of the outside part of the pipeline system, where the main measurement region instrumented with the optical fiber was located.

The presented study investigates feasibility of detection of small leaks in gas pipelines using fiber-optic DAS with optical fiber applied directly to the pipe surface. All the experiments were performed on a model pipeline setup built at a test site for technical safety of Federal Institute for Materials Research and Testing (BAM). The linear pipeline is partially located within the facility building from where it continuously extends through the building wall to the outside region. The pipeline consists of thirteen DN100 pipe segments connected together through flanges. Figure 1a depicts the schematic illustration of utilized pipeline setup, whilst a photo of the outside part of the pipeline is provided in Figure 1b. Except segment n. 8 which has only 2 m, all segments are 3 m long, giving the pipeline overall length of 38 m. Four individual pipe segments were instrumented with optical fiber for DAS measurement; three adjacent pipe segments (n. 9–11) of the outside part of the pipeline constitute the main measurement region and one segment (n. 2) close to the inside end of the pipeline is used as a remote reference region. Full fiber application on the entire pipeline was not feasible due to practical reasons. For the purposes of data evaluation, the reference pipe segment (n. 2) will be referred to as zone 0 and three outer instrumented pipe segments (n. 9–11) will be referenced as zones 1–3 from now on (Figure 1a).

The individual pipe segments have side adapters welded to them every 30 cm. Leak in the pipeline was simulated using holey adapter caps inserted in the side adapter located in the middle of the pipe zone 2, i.e., in the middle of the main DAS-monitored region (Figure 1a). Side adapter caps with circular holes of different diameters in 1–8 mm range were used. Both ends of the pipeline were closed and auxiliary pressure buffer was connected to the inside end of the pipeline through a 1-inch hose with remotely-controllable valve. The buffer could be pressurized up to 30 bars using compressed air. After the holey adapter of desired size was installed into the pipeline, the valve between the buffer and the pipeline was opened, leading to rapid pressurization of the pipeline and simulated leaking through the holey adapter. Pipeline and buffer internal pressures were monitored using electronic pressure gauges.

### 2.2. Employed Sensors

**Figure 2:**
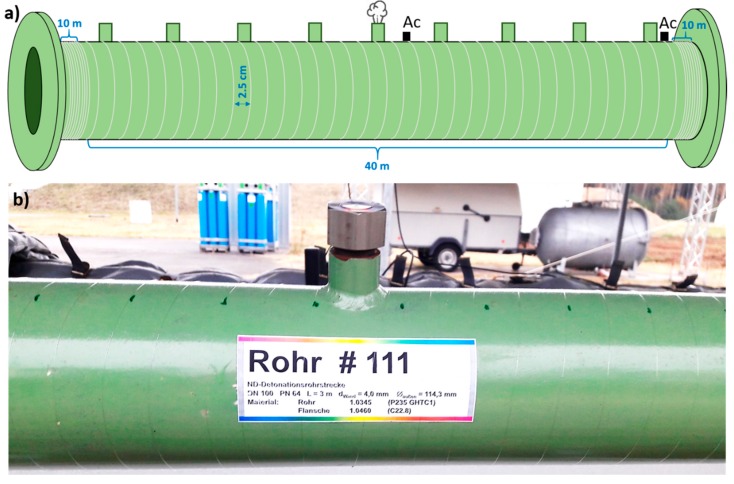
(**a**) Schematic illustration of the employed fiber application approach relying on fiber helical wrapping around the pipe segments. Position of simulated leak and reference accelerometers “Ac” (only in pipe zone 2) is indicated as well; (**b**) Detail photo of one of the instrumented pipe segments showing a pipe side adapter and applied fiber.

Standard single-mode optical fiber (SMF-28e, Corning, NY, USA) without any jacketing was used in this work. Figure 2a schematically illustrates fiber application approach used to instrument selected pipe segments with the optical fiber. The same fiber application approach is used for all four instrumented pipe segments. The fiber was wrapped helically around the main body of a pipe segment using a relatively small pitch of approximately 2.5 cm. A photo of one of the instrumented pipe segments showing helically applied fiber is presented in Figure 2b. Compared to simple straight fiber application along the pipe, helical wrapping increases fiber-to-pipe coverage ratio $R=L_{f}/L_{p}$, where $L_{f}$ is the length of the fiber used to cover the pipe section of length $L_{p}$. This helps to increase measurement sensitivity and spatial resolution for potential weak signals [40,41]. Considering pipe outer diameter and fiber application pitch, roughly 40 m of the fiber was applied to the main body of a 3 m-long pipe segment. In addition to these 40 m of the fiber, auxiliary 10 m of closely wound fiber were applied at the both ends of a pipe segment, close to the flanges. As the continual fiber application was not feasible due to the pipeline flanges, there is always a short freely-hanging fiber section between the pipe segments. These freely-hanging sections of the fiber proved to be prone to recording spurious signals. DAS systems essentially measure vibration signals effectively integrated over a fiber length proportional to the used interrogating pulse length. This may lead to “optical leaking” of the spurious signals from the freely-hanging fiber sections to the fiber applied on the pipe, especially if longer DAS pulse settings are used. The auxiliary fiber zones help to isolate the rest of the fiber applied to the pipe from the potential spurious signals coming from the freely-hanging fiber sections. Only data from the fiber sections applied to the to the relevant pipe zones is evaluated. All other interconnecting fiber sections are considered so-called dead-zones and signals coming from these sections are discarded in the evaluation. 

Commercial DAS system (Helios DAS, Fotech Solutions, Church Crookham, UK) is employed in the study for the distributed vibration measurement. The system represents the simplest amplitude-based implementation of the DAS technology. As discussed earlier, designation DAS is, in fact, a misnomer for this type of system as it offers only qualitative distributed vibration measurement. Nevertheless, we will continue to use this nomenclature in the paper as it seems to be established in the field and the used system is sold under this name. All presented measurements were performed using 200 ns laser pulse length and 80 kHz pulse repetition rate. Relatively large pulse length helps to increase system sensitivity for potential weak leak-induced signals. On the other hand, long pulse length $T_{p}$ limits the achievable DAS spatial resolution $r≅cT_{p}/\left(2n_{eff}\right)$, where c is the speed of light in vacuum and $n_{eff}$ is the effective refractive index of the optical fiber mode. Pulse length of 200 ns corresponds to roughly 20 m spatial resolution in the fiber. The lower DAS spatial resolution using long pulse lengths is compensated by fiber application with high fiber-to-pipe coverage ratio. For our fiber application, 200 ns pulse in the helically applied fiber corresponds to measurement spatial resolution of roughly 1.6 m long pipe section. This might not be sufficient to perform precise signal localization within the same pipe segment, however, localization of the signals between the different pipe segments should be possible.

In accordance to Nyquist theorem, the pulse repetition rate determines the maximal frequency of detectable vibration signals. Pulse repetition rate close to the maximal possible system setting was used in our measurements in order to cover the broadest possible spectral interval of potential vibration signals. At the same time, however, pulse repetition rate $f_{p}$ is inversely proportional to the maximal monitoring distance $L_{m}=c/\left(2n_{eff}f_{p}\right)$. Pulse repetition rate of 80 kHz corresponds roughly to 1.25 km maximal monitoring distance. This does not pose any limitation for our experiment where only relatively short fiber distance of roughly 250 m is monitored.

In addition to optical fiber, pipe segment with the leak (zone 2) was also instrumented with reference accelerometers (KS95B.100, MMF, Radebeul, Germany). The accelerometers were glued to the pipe surface from the top side; one in the middle of the segment close to the simulated leak and one close to the segment end (Figure 2a).

### 2.3. Experimental Methodology

The experiment consisted of a series of measurements taken at various combinations of leak sizes and pipeline internal pressures. For each leak size, a series of measurements was performed at gradually decreasing pressure. For each measurement series, the pressure buffer was firstly pumped up to 25–30 bars. When the desired buffer pressure level was reached, the valve between the buffer and the main pipeline was opened. After a brief period, pressures in the buffer and the pipeline equalized and a relatively steady leak from the holey adapter was achieved. Measurements with duration of 30 s were taken both with the DAS and reference accelerometers at selected pressure levels as the pipeline pressure was gradually decreasing to atmospheric pressure due to the leakage. As the result of constant leakage, the internal pressure during 30 s measurements does not remain unchanged. Table 1 lists approximate pressure intervals at which the individual measurements for different leak sizes were performed. For brevity, rounded nominal pressure values (as indicated in Table 1) will be used to reference the individual measurements throughout the paper.

The saved DAS raw data represents 2D matrix containing recorded optical intensity signals presented along two axes: measurement time and position along the fiber. Short-time fast Fourier transform (STFFT) was then applied to the data from individual position bins. Time-series data of each position bin were split into consecutive time windows containing *N* time measurement points and their spectral representation was calculated using fast Fourier transform (FFT). No windowing was used in the performed STFFT, however, the signals in each time window were detrended before FFT to minimize the impact of DC fluctuations of DAS signal on calculated spectra. FFT window length *N* of 8192 points was used for all the presented results. As discussed earlier, only data from the four relevant pipe zones are evaluated. Signals coming from the dead-zones are discarded. The prepared STFFT spectral evolution data of all relevant position bins represent the base for further signal evaluation and presentation in this paper. In general, three different types of signal spectral representation will be used in this paper:

- Time-averaged spectra—STFFT spectra from all individual STFFT time windows are averaged, separately for each position bin.
- Position-averaged spectra—STFFT spectra of all position bins belonging to the same pipe zone are averaged, separately for each STFFT time window.
- Overall zone spectra—STFFT spectra for a single pipe zone are averaged both over all the bins belonging to the zone and all the STFFT time windows.

All the zones have same size of 36 bins to keep the results of individual zones comparable. For all the spectral representations, a noise floor was subtracted from the individual spectra. Noise floor was estimated as a median value of the respective spectrum.

A similar STFFT spectra time-averaging approach that was used for the DAS data was also applied to the accelerometer data. Accelerometer data was captured with 200 kHz acquisition rate and FFT time window length of 32,768 points was used in the evaluation.

## 3. Results

**Figure 3:**
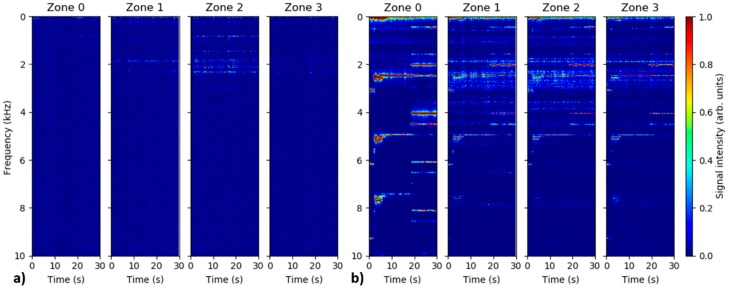
Temporal evolution of position-averaged distributed acoustic sensing (DAS) signal spectra in the individual pipe zones recorded for 1 mm leak at 10 bars (**a**) and 8 mm leak at 20 bars (**b**). Note that amplitude of spectra for 1 mm leak at 10 bars are scaled by factor 5 relatively to the spectra for 8 mm leak at 20 bars.

All DAS measurements were performed using 80 kHz (close to the maximal possible) pulse repetition rate in order to cover the maximal possible spectral range of potential vibration signals. Nevertheless, virtually no relevant signals were observed at frequencies above 10 kHz throughout the experiment. Therefore, no attention is paid to the spectral range above 10 kHz in this work. Numerous measurements for different combinations of simulated leak sizes and pipeline internal pressures listed in Table 1 were performed. Nevertheless, the results presented here are limited to the case of small leak rates, i.e., measurements at small leak sizes and/or low pressures. On one side, this is in accordance with the original motivation of this work, where detection of pinhole leaks represents the addressed technological challenge. On the other side, it is also a consequence of inherent limitations of our measurement setup. In our pipeline system, high leak rates lead to the appearance of strong parasitic vibration signals originating from the internal air-flow through the buffer-pipeline interconnecting hose. These strong vibrations propagate along the pipeline and may remain detectable by the DAS system even in the zone 3, more than 20 m away from the buffer-pipeline interconnection. This negative effect is illustrated in Figure 3 comparing the temporal evolution of position-averaged DAS signal spectra in the individual pipe zones for two separate measurements; 1 mm leak at 10 bars and 8 mm leak at 20 bars. These two measurements represent two extremal experimental condition cases in terms of leak rates.

Figure 3a, presenting a low leak rate case, shows the presence of signal consisting of a collection of weak but spectrally distinct frequencies predominantly located in the spectral range below 2.5 kHz. The signals are dominant in the pipe zone 2 where the simulated leak is located, while no signals are observed in the reference zone 0. This indicates that the observed features are actual leak-induced vibration signals detected by the DAS system. The spectrum of detected signal remains relatively stable over the measurement time of 30 s. On the other hand, more complex and temporally evolving spectra are observed in Figure 3b presenting a large leak rate scenario. The strong parasitic signals originating from the buffer-pipeline interconnection are most apparent in zone 0, but are also detectable in all other pipeline zones. Focusing on the spectrally distinct signals in zone 0, only the features below 4 kHz represent actual vibration signals. The rest of the spectral features visible in the graph at higher frequencies are the harmonics of these vibration signals. The appearance of the higher harmonics for strong signals is a well-known and inherent problem for amplitude-based DAS systems. The higher-harmonic signals do not bare any relevance to the experiment, nevertheless, they can obscure actual signals and disallow their successful detection and interpretation. The appearance of the parasitic vibration signals at the buffer-pipeline interconnection is highly detrimental for the performed experiment and disallows us to perform reliable evaluation for the large leak rates. Therefore, we focus further predominantly on the evaluation of the small leak rate cases, i.e., measurements at small leak sizes and/or low pressures. For the evaluated measurement cases, the signals were predominantly located in the frequency range below 5 kHz. Therefore, only this spectral range will be further included in the presented evaluation. 

**Figure 4:**
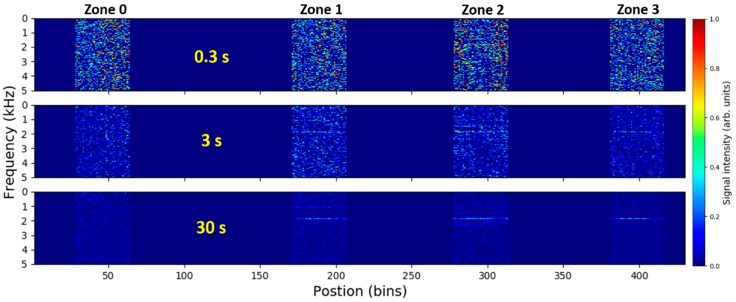
Time-averaged DAS signal spectra along the fiber recorded for 1 mm leak at 5 bars and averaged over different acquisition times. Signals from fiber dead-zones between the monitored pipe zones are discarded, i.e., set to zero. Label acquisition times of 0.3 s, 3 s and 30 s correspond to spectral averaging over 3, 29 and 292 short-time fast Fourier transform (STFFT) windows.

As illustrated in Figure 3a, leak-induced signals at lower leak rates are temporally stable. This allows us to perform temporal-averaging of the recorded signals in order to increase signal-to-noise ratio (SNR) and detectability of weak signals. Figure 4 depicts the time-averaged DAS signal spectra along the fiber for 1 mm leak at 5 bars. As discussed before, signals from fiber dead-zones are discarded, i.e., set to zero. Three different acquisitions times, over which the signal spectra are averaged, were used. The used acquisition times were roughly 0.3 s, 3 s and 30 s of the measurement time. The selected acquisition times corresponds to spectral averaging of 3, 29 and 292 STFFT windows, respectively. The figure illustrates how the temporal averaging can help to improve detectability of weak but temporally-stationary signals from small pipeline leaks. While no detectable signals are visible for 0.3 s acquisition time, still weak but clearly detectable leak-induced signal can be observed when the spectra from the entire measurement interval of 30 s were averaged. All spectral representation of the data presented here from now on were prepared using discussed time-averaging over the entire 30 s duration of the individual measurements.

**Figure 5:**
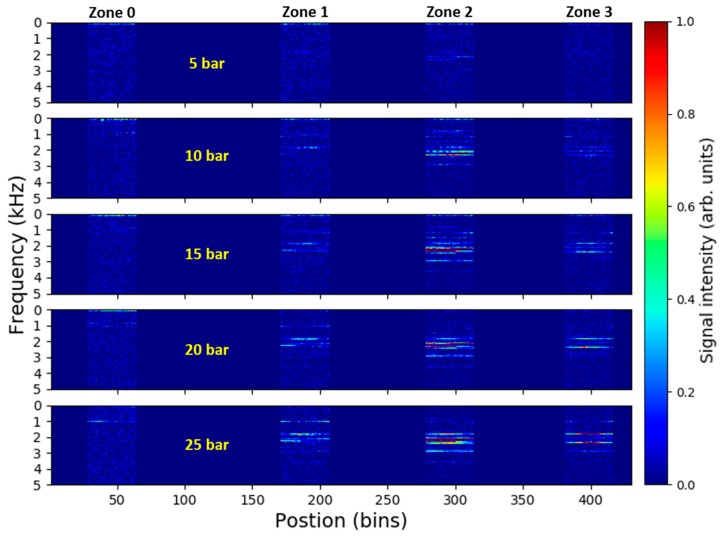
Time-averaged DAS signal spectra along the fiber recorded for 1 mm leak at different pressure levels. Signals from fiber dead-zones between the monitored pipe zones are discarded, i.e., set to zero.

Figure 5 shows the time-averaged DAS signal spectra along the fiber for the measurement series of 1 mm leak at different pressures. Leak-induced vibration signals comprising of multiple distinct spectral components are clearly visible in the pipe zones 1–3 for all tested pressure levels. On the other hand, virtually no detectable signals are present in the remote reference zone 0. The magnitude and complexity of the signals increase with increasing pipeline pressure. In addition, it appears that the strongest signal is present in the zone 2 where the simulated leak is located. This all indicates that the recorded signals are associated with the leak-generated vibrations and might be used as an indication for pipeline leakage detection and localization. 

**Figure 6:**
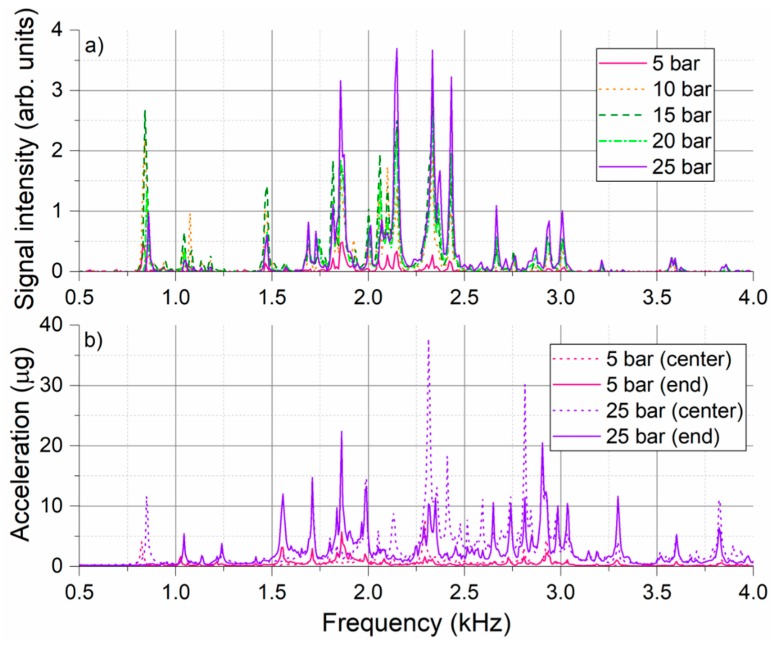
Signal spectra recorded in the pipe zone 2 for the measurement series of 1 mm leak at different pressure levels; (**a**) Overall (time- and position-averaged) DAS signal spectra from the pipe zone 2; (**b**) Time-averaged signal spectra from the reference accelerometer placed in the middle of the pipe zone 2 close to the leak (center) and at the edge of the pipe zone close to the flange (end).

To further analyze the spectral character of recorded signals, presented time-averaged data were additionally averaged over all the bins corresponding to the individual pipe zones. Such overall signal spectra recorded for 1 mm leak measurement series in the pipe zone 2 are presented in Figure 6a. Spectra of signals recorded by the two reference accelerometers are included in Figure 6b for comparison. Only measurements at the lowest (5 bars) and highest (25 bars) tested pressure levels are included for accelerometer data for the sake of graph readability. Figure 6a shows that the overall shape of the DAS leak-induced signal spectrum remains relatively stable. All spectral features that can be recognized in the lowest (5 bars) pressure measurement, remain in the signal spectrum also for all other measurements. Some new spectral features appear in the spectrum at higher pressure levels. However, this is most likely associated with the general increase of signal amplitude which “uncovers” the peaks that were before below measurement detection level, i.e., had too low SNR. Spectral position of the majority of dominant peaks is stable. Nevertheless, slight frequency shifts are observed for some of the spectral features, e.g., leftmost peak that shifts gradually from 830 Hz to 860 Hz with increasing pipe pressure.

Comparison between the accelerometer in the middle and at the end of the pipe segment shows that while some frequency components are common to both curves, there are also several complementary frequencies that appear only in one of the spectra. For example, the leftmost peak around 850 Hz appearing only in the “central” accelerometer signal and the peak around 1.85 kHz appearing only in the “end” accelerometer signal. In this respect, displayed DAS data represents position-averaged spectra. This is not only due to the numerical averaging of the spectra from the zone 2 performed in the evaluation step, but also due to inherent “optical integration” of the signals from the finite length of the fiber proportional to the utilized DAS pulse length. For our fiber application, 200 ns pulse in the helically applied fiber corresponds to DAS spatial resolution of roughly 1.6 m long pipe section. Nevertheless, there is a good agreement between the signals collected by the DAS system and reference accelerometers. Similarly, as for the DAS data, accelerometer results confirm that the general shape of the signal spectrum remains largely the same and only its amplitude increases with increasing pipe pressure. Exceptions are certain spectral features which shift slightly with the pressure. This shift has been observed consistently with the DAS system and the accelerometers, e.g., positive shift of 850 Hz spectral peak with rising pressure. The position of all dominant spectral peaks agrees well between the two measurement technologies, however, the relative amplitude of the individual peaks may differ considerably. This may be caused by the different sensitivities of the sensors to the vibration signals of different nature (different pipe vibration modes) present in the experiment. Spectral dependence of signal transfer from the pipe to the fiber may also play a role. Accelerometer data generally contain strong leak-generated vibration signals up to 50 kHz (not shown here), while almost no leak-generated signals above 5 kHz can be detected with the DAS system. Since no spectral-dependence of the DAS system sensitivity is known, the effect is most likely associated with the mechanical transfer of pipe vibration signals to the sensing fiber. It is also important to remind that, unlike accelerometers, utilized DAS system does not have a linear and time invariant transfer function and, therefore, does not provide a true measurement of signal amplitudes.

**Figure 7:**
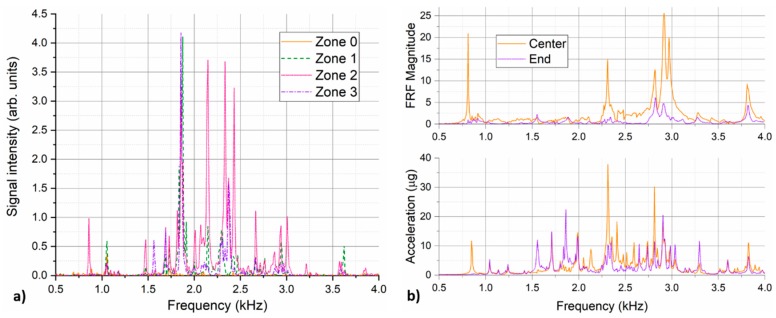
(**a**) Overall DAS signal spectra in different pipe zones for 1 mm leak at 25 bars; (**b**) Comparison of accelerometer signals recorded in the pipe zone 2 for 1 mm leak at 25 bars (bottom) and pipe frequency response function (FRF) determined by impact hammer test (top).

Figure 7a shows the overall DAS signal spectra from all four pipe zones for the case of 1 mm leak at 25 bars. Virtually no signals are observed in the reference zone 0. Among three pipe zones in the monitored region, the signals are clearly dominant in the pipe zone 2, where the simulated leak is located. This is an indication that the recorded signals originate from the leak. Some of the spectral features seem to spread to the neighboring pipe segments more efficiently, e.g., spectral peak around 1.85 kHz. Other spectral features appear to remain strongly localized in the pipe segment with the leak, e.g., spectral peak around 850 Hz. To help to understand the nature of the observed vibration signals, pipeline natural frequencies were investigated using impact hammer testing [42]. The pipeline was excited with an impact hammer (ICP^®^ 086C03, PCB Piezotronics, Depew, NY, USA) on the top of the pipeline side adapter where the leak was simulated and the pipeline response was measured using accelerometers glued in the various positions on the pipeline. Pipeline frequency response function (FRF) was determined from 20 individual hits. Figure 7b compares the signal spectra collected by the two reference accelerometers in the pipe segment 2 during the experiment with 1 mm leak at 25 bars and the pipeline FRF determined for the two accelerometers with the similar placement as in the leak experiment. Two graphs show a good agreement for the majority of the dominant peaks, revealing that the observed signals correspond to the pipeline eigenfrequencies excited by a broadband excitation (white noise) generated by a pressurized air escaping the pipeline through the small leak. Depending on the nature of the vibration mode, some of the modes may propagate along the pipeline more efficiently while others remain localized only to the immediate vicinity of the excitation source. With regard to comparison of DAS and accelerometer data, one has to note that accelerometers are predominantly sensitive to the vibrations in direction of their sensitivity axis (in our case vertical direction), while DAS is sensitive to all vibration signals that lead to straining of the helically-applied fiber. These could be different transversal, longitudinal and circumferential pipe vibration modes.

**Figure 8:**
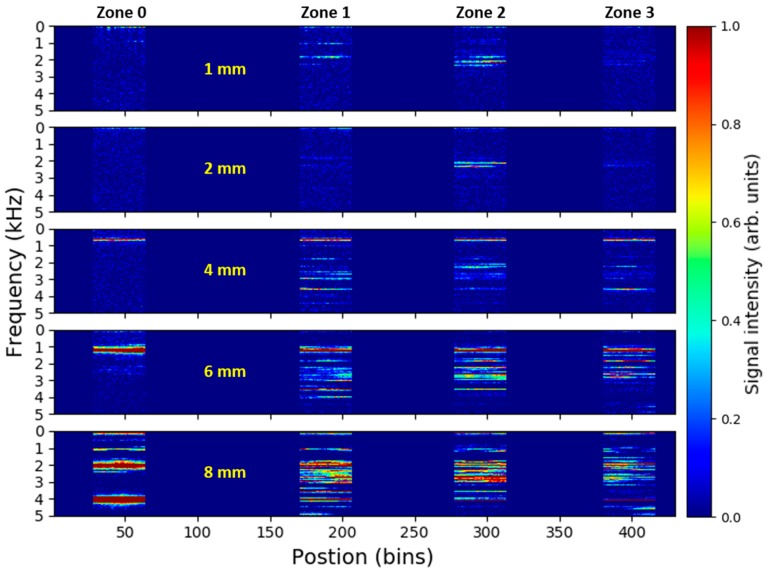
Time-averaged DAS signal spectra along the fiber recorded for different sizes of simulated leak at 10 bars. Signals from fiber dead-zones between the monitored pipe zones are discarded, i.e., set to zero.

In analogy to Figure 5, Figure 8 depicts the time-averaged DAS signal spectra along the fiber for measurement series of different size leaks at the same pressure level of 10 bars. The figure shows that the spectrally-distinct leak-induced signals are detectable in pipe zones 1–3 also for larger diameters of simulated leaks and might still be used as pointers for detection and localization of the pipeline leak. Nevertheless, due to limitations of our pipeline setup discussed earlier, strong parasitic signals originating from the buffer-pipeline interconnection and propagating along the entire pipeline appear in the measurement for larger leak sizes. For the presented measurement series, such a parasitic signal spanning through all the monitored pipe zones is apparent starting from 4 mm leak size, where it is visible in a form of a sharp spectral feature around 700 Hz. With increasing leak size, the amplitude of the parasitic signal rises and its frequency increases as well. The spectra with the highest complexity is generally observed in the pipe zone 2 where the leak is simulated. The complexity and strength of the leak-induced signals seem to increase with rising leak size. 

**Figure 9:**
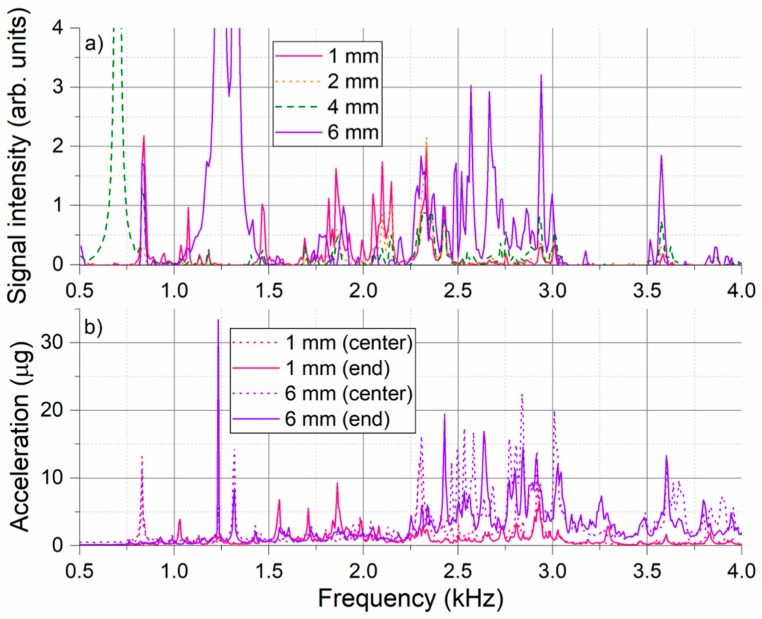
Signal spectra recorded in the pipe zone 2 for the measurement series with different size leaks at the same pressure level of 10 bars; (**a**) Overall (time- and position-averaged) DAS signal spectra from the pipe zone 2; (**b**) Time-averaged signal spectra from the reference accelerometer placed in the middle of the pipe zone 2 close to the leak (center) and at the edge of the pipe zone close to the flange (end).

The overall DAS signal spectra from the pipe zone 2 for the measurement series presented in Figure 8 are depicted in Figure 9a. For 8 mm leak, the parasitic signal gets strong enough to lead to appearance of its harmonic frequencies which disallows reliable signal evaluation. Therefore, data from this measurement is not displayed here and is also omitted from further evaluation. For reference, Figure 9b shows the accelerometer signal spectra recorded for the same measurement series. Only measurements for the smallest (1 mm) and the largest included (6 mm) leak size are displayed for the sake of graph readability. Compared to the pressure increase at constant leak size, that seems to increase only magnitude of the detected spectral signals, increase of leak size at constant pressure appears to have a more profound impact on general spectral shape of the detected signal. This is not so much in terms of changing position of individual sharp spectral features, but in terms of excitation efficiency of spectral components in different spectral bands. Increasing leak size at constant pressure level seems to lead to excitation of pipe vibrations at higher frequencies. This is most apparent comparing the presented DAS spectra for two extremal cases, i.e., for 1 mm and 6 mm leak. For 1 mm leak, recorded spectrum is dominated by spectral features below 2.5 kHz, while features above 2.5 kHz are clearly dominant for 6 mm leak. This trend is also confirmed by the accelerometer data. Again, good general agreement between the DAS and accelerometer data can be found with majority of dominant peaks visible in both presented graphs. Appearance of parasitic frequency peaks around 1.23 kHz and 1.32 kHz is notable in the accelerometer data for the 6 mm leak as well.

The presented results show that the DAS system is capable of measuring local pipeline vibrations induced by the pipeline leak. These signals detectable by the DAS system using sensing fiber applied directly to the pipeline may serve as the pointers for the detection and localization of the pipeline leaks. Post-processing and interpretation of the measured DAS data to provide reliable and comprehensible pipeline status report to the operators constitutes a crucial part of DAS-based pipeline monitoring systems. These tasks typically rely on various pattern recognition and machine learning methods which have been the subject of intense research over the last years [43]. This paper focuses on investigation of the physical capability of DAS to detect weak leak-induced pipeline vibrations. Detailed contemplation of the most suitable detection and localization strategy goes beyond the scope of this paper. Nevertheless, we present rudimentary leak localization approach based on simple spectral integration of the time-averaged DAS signals in frequency domain. 

**Figure 10:**
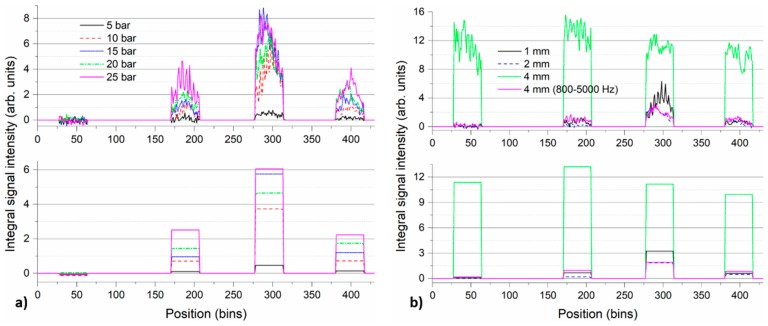
Integral spectral intensity of the detected DAS signals along the fiber for the measurement series with 1 mm leak at different pressure levels (**a**) and for the measurement series at constant pressure level of 10 bars and different leak sizes (**b**). The time-averaged spectra of DAS signals from individual measurements were integrated in 500–5000 Hz range. Additional curve for 4 mm leak at 10 bars using spectral integration in 800–5000 Hz range is included in plot (**b**). Top-row graphs show spectrally-integrated signals for individual bins in the four pipe zones. Bottom-row graphs show corresponding zone-averaged signal integral spectral intensities.

The detected signal predominantly represents various pipeline vibration modes excited by the broadband white noise signal generated by the leaking medium. The detected signal is composed of a collection of spectrally distinct components broadly located in the spectral range between 500 Hz and 5 kHz. We showed that the position and amplitude of the individual spectral features may change slightly depending on the leak parameters. In addition, these features represent eigenfrequencies of the pipeline which may also change depending on the particular installation and geometry of the pipeline. Rather than spectral filtering and tracking of a single frequency feature (vibration mode), considering integral intensity in the entire spectral band may be a more appropriate approach for robust leak detection and localization. Figure 10 depicts the signal integral spectral intensity along the fiber for the measurement series with 1 mm leak at different pressures (a) and for the measurement series with various leak sizes at constant pressure of 10 bars (b). Top-row graphs were prepared by spectral integration of the individual time-averaged DAS signal spectra evolutions along the fiber presented in Figure 5 and Figure 8, respectively. Spectral integration was performed over 500–5000 Hz spectral range, individually for each bin along the fiber. Bottom graphs present zone-averaged integral spectral intensity, where the curves presented in the top-row graphs are further averaged over all the bins corresponding to the individual pipe zones.

Figure 10 illustrates the ability of the proposed approach to indicate the presence and localize the leak within the pipe zone 2 where it was simulated. For the measurement series with 1 mm leak (Figure 10a), displayed curves indicate the presence of leak-induced signals in zones 1–3, while virtually no signals are present in the reference zone 0. The dominance of the integral signals in the zone 2 is obvious for all pressure levels and provides good leak localization performance. Position averaging of the integral spectral intensity data (bottom-row graph) may help to increase localization ability of the approach also for the case of small leak rates (1 mm at 5 bars) at the price of decreased spatial resolution. As discussed earlier, situation for the measurement series at constant pressure of 10 bars with increasing leak sizes (Figure 10b) is more complicated due to the appearance of the parasitic signals from the buffer-pipeline interconnection. Figure 10b shows that the leak localization within zone 2 is possible using the proposed approach for lower leak sizes (1 mm and 2 mm). However, already for 4 mm leak, the evaluation is impaired due to the presence of the strong parasitic signal at around 700 Hz, which falls within the spectral band of interest (500–5000 Hz). The issue can be partly alleviated by spectral discrimination of the parasitic signals. Figure 10b also contains a curve for 4 mm leak at 10 bars where the spectral integration only in 800–5000 Hz spectral band was performed, thus avoiding the impact of the parasitic signal. With this adjusted strategy, localization of 4 mm leak within the pipe zone 2 is possible as well. The post-processing and treatment of the parasitic signals go beyond the scope of this work. These measurement artefacts represent limitations of our experimental setup rather than limitations of the presented measurement approach and are not expected to occur in realistic pipeline systems.

**Figure 11:**
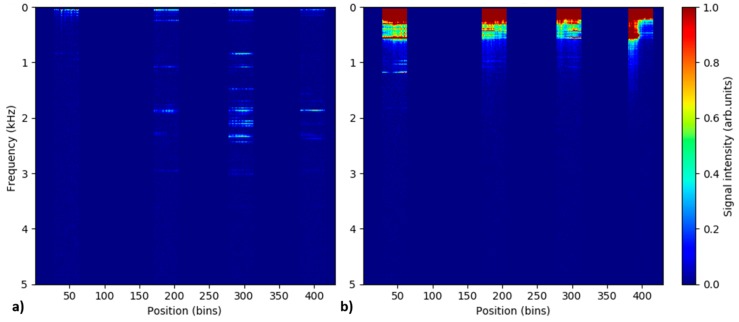
Time-averaged DAS signal spectra along the fiber recorded for 1 mm leak at 10 bars (**a**) and for airflow through the leak-free pipeline at atmospheric pressure and flow speed of 70 m/s (**b**). Signals from fiber dead-zones between the monitored pipe zones are discarded, i.e., set to zero.

In reality, leak detection systems have to operate under the presence of various background vibrations that might obscure the leak-induced signals. Most obvious source of background signals are the pipeline vibrations induced by the flow of the medium itself. Our current pipeline system does not allow performance of leak detection experiments at simultaneous medium flow through the pipeline. Nevertheless, a separate rudimentary evaluation of flow-induced vibrations in our pipeline was performed. For the throughflow measurement, both ends of the test pipeline were opened and a large industrial air blower was connected to the inside end of the pipeline (at the side of zone 0) through a large-diameter rubber hose. Airflow through the leak-free pipeline at various flow speeds was simulated by varying the blower rpm setting. Figure 11 compares the signals recorded for the test pipeline system for the case of 1 mm leak at 10 bars and flow-induced vibrations at airflow speed of 70 m/s. Exactly same time-averaging approach and evaluation algorithms were used for the presented leak and throughflow measurements. Compared to weak leak-induced vibrations, gas flow through the pipeline may induce considerably stronger vibrations. These, however, predominantly occupy low-frequency end of the spectrum. Even for the unrealistically high flow speed of 70 m/s (gas pipeline maximal flow speeds typically at the level of 20 m/s), measured vibrations are largely contained to the spectral range below 500 Hz. Since the vast majority of leak-induced signals observed in our experiments is located above 1 kHz, proposed leak detection approach may provide good inherent isolation from these common background signals induced by the medium flow. Moreover, the amplitude of the flow-induced signals decreases rapidly with diminishing flow speed (data not presented here). This further decreases the severity of the issue at realistic flow rates. At the same time, however, we have to acknowledge that the airflow measurements were performed without internal pipeline pressure, which may have an impact on amplitudes of generated flow-related vibrations. 

## 4. Discussion

Majority of DAS pipeline monitoring systems typically focus only on lower-frequency signals (hundreds of Hz) in order to extend the system monitoring distance. The maximal spectral limit of the DAS measurement (pulse repetition rate) is inversely proportional to the maximal achievable monitoring length. In our case, virtually all measured signals representing pipeline natural vibrations had frequencies in range between 0.5 kHz and 5 kHz. This means that the DAS pulse repetition rate of 10 kHz would be required to perform the measurement. Monitoring at 10 kHz pulse repetition rate corresponds to the DAS measurement range of roughly 10 km, limiting this approach to monitoring of short- to medium-length pipelines. On the other hand, focusing only on signals in 0.5–5 kHz range can potentially provide good isolation from background vibration signals that usually occupy low-frequency range (Figure 11). These might be signals generated by the medium flow through the pipeline or other external sources associated with pipeline operation or surrounding environment (e.g., traffic). At the same time, the presented approach is based on the temporal-averaging of continuous leak-generated signals. This, in turn, provides a certain degree of inherent filtration of short-term (transient) noise signals. 

In this work, we used fiber helical wrapping with fairly small pitch of 2.5 cm in order to maximize fiber-to-pipe coverage ratio R, and thus increase system sensitivity for potentially weak and highly-localized leak signals. Comparison with reference accelerometer data (e.g., Figure 6) shows that pipeline vibrations with acceleration values down to single µg are detectable in performed experiment. At the same time, however, this fiber application approach increases instrumentation complexity and decreases system ultimate monitoring range, i.e., length of the pipeline that can be covered/monitored with the fiber of given length. The presented results (e.g., Figure 10) showed that the leak-induced signals partially spread from the pipe zone 2 with the leak into the neighboring zones 1 and 3. This is a consequence of propagation of the leak-induced vibrations along the pipeline. Nevertheless, this propagation is subject to relatively high damping/attenuation and the leak-induced signals are detectable only on a limited range. The spreading of the leak-induced signals along the pipeline would limit spatial resolution of leak localization to a few meters. This is, however, still very attractive and acceptable spatial resolution which is common for other DAS pipeline monitoring systems as well. On the other side, vibration spreading partly relaxes the requirements on the fiber application. Fact that the generated signals are detectable not only in the immediate vicinity of the leak, but also spread-out over few meters around the leak suggest that also helical application with larger pitch (lower R) or even simple linear application can be used. This would help to simplify and speed-up the fiber application procedure as well as to extend system monitoring range. One has to note that changes in application approach might impact detection efficiency for different types of pipeline vibration modes as some of them may fail to efficiently induce fiber straining.

Origin and signature of pipeline leaks can differ considerably depending on a number of factors including transported medium, pipeline type and operation conditions. Therefore, the discussion of optimal leak detection approach represents a complex issue. Pipeline leak detection systems typically need to be tailored and verified for the individual application cases. Presented work targets fundamental investigation of DAS capabilities for detection of pinhole leaks in gas pipelines. In this context, performed experiment represents only an initial, highly-idealized case. Some of the crucial questions for further consideration of the presented approach include:

- How much does the pipeline natural vibration spectrum change for various pipeline types and installations?
- How crucial is the influence of additional vibration damping for underground or underwater pipelines?
- How does particular fiber application approach influence the system sensitivity (transfer function) for different pipeline vibration modes?
- How the particular geometry of the leak influences generated vibration signals.
- Can the approach be transferred also to liquid- and mixed-phase pipelines?

Nevertheless, we showed that using the direct helical fiber application on the pipe, DAS system is capable of detecting weak leak-induced pipeline vibrations. Assuming a realistic flow speed of 10 m/s through our DN100 pipeline, the smallest successfully detected leak (1 mm at 5 bars) corresponds to a leak rate of roughly 0.14% of the volume flow. This is extremely small value typically unattainable with the majority of common leak detection systems.

## 5. Conclusions

Presented work investigates the feasibility of pinhole leak detection in gas pipelines using fiber-optic distributed acoustic sensing. A fiber application approach relying on direct fiber wrapping around the pipeline is used to detect weak leak-induced pipeline vibrations. Reference accelerometer measurement is used to analyze the nature of recorded vibration signals. The results reveal that DAS measurement using direct helical fiber application is capable of detecting pipeline natural vibration modes induced by the broadband leak-noise excitation. In the performed experimental campaign, pipeline vibration modes with acceleration values down to single µg were detected. Influence of pipeline internal pressure and leak size on the leak-induced vibrations was studied. Increase of pipeline pressure at constant leak size was shown to lead to an increase of magnitude of the induced signal while its general spectral content remains relatively stable. On the other hand, leaking through holes of increasing sizes leads to excitation of vibrational modes with higher frequencies. Simple leak detection approach based on spectral integration of time-averaged DAS signals in frequency domain (0.5–5 kHz) was proposed. Potential advantages and limitations of the presented monitoring approach with regard to its practical applicability were discussed. The approach was shown to be capable of detection and localization of gas pipeline leaks with leak rates well below 1% of the pipeline flow volume and might be of interest for short- to medium-length gas pipeline systems.

## Figures and Tables

**Table 1:** Intervals of real pipeline pressures (bars) at which the individual measurements were performed. Listed leak sizes and nominal pressure values will be used to reference the individual measurements throughout the paper.

Nom. Pressure\Leak Size	1 mm	2 mm	4 mm	6 mm	8 mm
**25 bar**	25.7–25.5	26–23.8	25–24	-	-
**20 bar**	20.5–20.2	21–19.9	20–19.1	20.7–19.4	21–18.1
**15 bar**	15.5–15.31	15.5–14.5	15–14.35	15.5–14.3	16–13.9
**10 bar**	10.53–10.42	15.5–9.95	10–9.56	10.5–9.7	10.8–9.3
**6 bar**	-	6.15–5.8	6–5.73	6.3–5.8	6.4–5.55
**5 bar**	5.7–5.6	-	-	-	-
**4 bar**	-	4.15–3.93	4–3.82	4.15–3.8	4.3–3.7
